# Phase homogeneity in ternary amorphous solid dispersions and its impact on solubility, dissolution and supersaturation – Influence of processing and hydroxypropyl cellulose grade

**DOI:** 10.1016/j.ijpx.2023.100222

**Published:** 2023-12-02

**Authors:** Florian Pöstges, Jonas Lenhart, Edmont Stoyanov, Dominique J. Lunter, Karl G. Wagner

**Affiliations:** a Department of Pharmaceutical Technology and Biopharmaceutics, University of Bonn, Gerhard-Domagk-Str. 3, 53121 Bonn, Germany; b Department of Pharmaceutical Technology, Faculty of Sciences, University of Tübingen, Auf d. Morgenstelle 8, 72076 Tübingen, Germany; c Nisso Chemical Europe GmbH, Berliner Allee 42, 40212 Düsseldorf, Germany

**Keywords:** Ternary amorphous solid dispersions, Supersaturation, Polymer mixing, Hydroxypropyl cellulose, Confocal Raman spectroscopy, Dissolution

## Abstract

As performance of ternary amorphous solid dispersions (ASDs) depends on the solid-state characteristics and polymer mixing, a comprehensive understanding of synergistic interactions between the polymers in regard of dissolution enhancement of poorly soluble drugs and subsequent supersaturation stabilization is necessary. By choosing hot-melt extrusion (HME) and vacuum compression molding (VCM) as preparation techniques, we manipulated the phase behavior of ternary efavirenz (EFV) ASDs, comprising of either hydroxypropyl cellulose (HPC)-SSL or HPC-UL in combination with Eudragit® L 100–55 (EL 100–55) (50:50 polymer ratio), leading to single-phased (HME) and heterogeneous ASDs (VCM). Due to higher kinetic solid-state solubility of EFV in HPC polymers compared to EL 100–55, we visualized higher drug distribution into HPC-rich phases of the phase-separated ternary VCM ASDs via confocal Raman microscopy. Additionally, we observed differences in the extent of phase-separation in dependence on the selected HPC grade. As HPC-UL exhibited decisive lower melt viscosity than HPC-SSL, formation of partially miscible phases between HPC-UL and EL 100–55 was facilitated. Consequently, as homogeneously mixed polymer phases were required for optimal extent of solubility improvement, the manufacturing-dependent differences in dissolution performances were smaller using HPC-UL, instead of HPC-SSL, i.e. using HPC-UL was less demanding on shear stress provided by the process.

## Highlights

• Only hot-melt extrusion led to homogeneous ternary ASDs.
• Preferred drug distribution into HPC-rich phases of heterogeneous VCM ASDs.
• Lower melt viscosity of HPC-UL compared to -SSL facilitated VCM polymer miscibility.
• Miscible polymer phases were decisive for optimal dissolution.
• Lower manufacturing-dependent differences in dissolutions using the HPC grade -UL.

## Graphical abstract

**Figure:**
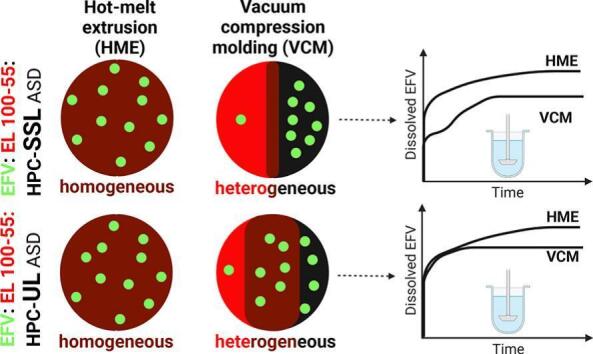

## 1 Introduction

The preparation of amorphous solid dispersions (ASDs) is one of the most common and popular formulation principles to overcome bioavailability limitations due to low solubility by generating and maintaining a supersaturated state of the drug in the dissolution (Bhujbal et al., 2021; Mishra et al., 2015; Pandi et al., 2020). During the ASD preparation process the poorly soluble drug is incorporated amorphously into a polymer matrix, mostly consisting of a single polymer (binary ASDs) (Chiou and Riegelman, 1971; Meng et al., 2015). To enable a complete amorphous stabilization and to prevent recrystallization/ phase-separation, the individual solid-state solubility of the drug of interest within the selected polymer must be considered. Immiscibility or the exceedance of the solubility of the drug would lead to the formation of amorphous or crystalline drug-rich phases (Alzahrani et al., 2022; Li et al., 2016; Sharma et al., 2021), and to a pronounced reduction of the supersaturation performance (Wlodarski et al., 2018). Regarding the solubility enhancement, an optimal dissolution of an ASD is described by a high initial dissolution rate, followed by an inhibition of precipitation and stabilization of the supersaturated state (Brouwers et al., 2009; Hanada et al., 2021). As similar to the solid-state stabilization generating and maintaining supersaturated drug solutions upon dissolution dependent on specific interactions between drug and polymer, the correct ASD-forming polymer needs to be selected carefully (Amponsah-Efah et al., 2021; Pöstges et al., 2023; Warren et al., 2010). Monschke et al. described Eudragit® L 100–55 (EL 100–55) as excellent matrix polymer for a ketoconazole ASD, as a high initial dissolution rate, followed by supersaturation stabilization for the entire observation period of 180 min, was observed in pH 6.8 medium (Monschke et al., 2021).

However, in some cases one single polymer cannot fulfill both requirements for being an optimal supersaturating ASD-forming polymer, as either a high initial drug release or an effective precipitation inhibition is missing upon dissolution (Alonzo et al., 2011; Xie and Taylor, 2016). Thus as alternative to the selection of one polymer, polymer combinations for forming ternary ASDs can be utilized, leading to synergistic enhancement in terms of solubility enhancement and supersaturation stabilization (Prasad et al., 2014; Zecevic et al., 2014).

By utilizing two polymers, a homogeneous or a heterogeneous ternary ASD can be obtained, depending on mixing of both polymers (Lyu et al., 2005). As the mixing of polymers can impact the intermolecular interactions between the polymers and the drugs, the phase behavior needs to be investigated (Butreddy, 2022; Sarpal et al., 2020). Monschke and Wagner processed a ternary ASD, consisting of polyvinyl alcohol (PVA) and hydroxypropylmethylcellulose acetate succinate (HPMCAS), and detected two glass transition temperatures (T_g_s), representing PVA-richer and HPMCAS-richer phases, thus these polymers were not miscible or only partially miscible. Although the processed ternary ASD outperformed the corresponding binary ASDs, the ternary ASD did not exhibit the full potential of each polymer, as the dissolution of the fast dissolving binary PVA ASD with externally added HPMCAS as precipitation inhibitor demonstrated superior dissolution performance (Monschke and Wagner, 2020). Instead, PVA was reported to be miscible with copovidone (PVP-VA) (30:70 polymer mass ratio). The ternary ASD using itraconazole as drug demonstrated superior dissolution performance compared to the corresponding binary ASDs (Wlodarski et al., 2018). Synergistic interplay of miscible polymer blends in terms of solubility enhancement was also observed for spray-dried ternary griseofulvin ASDs, using hydroxypropylmethylcellulose and methacrylic acid copolymers (Ohyagi et al., 2017).

Although the importance of homogeneous and miscible polymer blends were emphasized in several studies (Higashi et al., 2015; Marks et al., 2014; Thompson et al., 2023), ternary ASD processing was also shown to be beneficial when immiscible polymer blends were utilized (Hörmann et al., 2018; Six et al., 2004; Yang et al., 2013).

Yang et al. processed ternary ASDs, consisting of felodipine, Eudragit® E PO, and PVP-VA and observed phase-separation phenomena, as the polymers were not miscible and felodipine was preferably distributed within the PVA-VA-richer domains. However, compared to the binary ASD formulations, the ternary ASD (10% drug load) benefited in dissolution and stability due to overall improvement of physical properties, as felodipine demonstrated high solubility in the hydrophilic PVP-VA for exhibiting drug-polymer interactions, and Eudragit® E PO reduced the hygroscopicity of the formulation. Thus, for mechanistic understanding of the improved stability and dissolution phenomena, the investigation of the drug distribution in the phase-separated ASD was demonstrated to be decisive.

In a previous study of our workgroup, manufacturing-dependent polymer mixing was demonstrated. Placebo and ternary celecoxib (CXB) ASD formulations using the polymers EL 100–55 and hydroxypropyl cellulose (HPC)-SSL (50:50 polymer mass ratio) were prepared via vacuum compression molding (VCM) and hot-melt extrusion (HME). During VCM processing, the mixture was only exposed to heat, while the ASD preparation using HME occurred under heat and additional shear forces through the kneading elements. While VCM did not lead to a homogeneous polymer mixture, a single-phased system was obtained after the HME process. It was assumed that shear forces through HME were required for homogeneous mixing of these polymers. As a homogeneous and intimate polymer mixture was important for the synergistic enhancement of the supersaturation of the drug, the determination of the phase homogeneity and interactions of the polymers was highly relevant (Pöstges et al., 2022).

The aim of this current study was to extend the insights of the previous study and to gain deeper understanding of the impact of phase homogeneity/ interaction on dissolution performances of ternary EL 100–55: HPC ASDs, using efavirenz (EFV) as alternative model drug. Next to HPC-SSL (molecular weight of 40,000 g/mol), we introduced HPC-UL (molecular weight of 20,000 g/mol) as additional polymer partner of EL 100–55. Therefore, not only the dependence of the manufacturing technique (VCM and HME), but also the selection of the HPC grade on the solid-state and dissolution performance of ternary ASDs was investigated and evaluated. Additionally, the drug distribution within the phase-separated ternary ASDs and the underlying mechanism were determined, since the knowledge about the drug localization would provide mechanistic insights regarding solid-state solubilities and dissolution processes.

## 2 Materials and methods

### 2.1 Materials

HPC-SSL and HPC-UL were supplied by Nippon Soda Co., Ltd. (Tokyo, Japan). EL 100–55 was kindly donated by Evonik (Darmstadt, Germany). The model drug EFV was purchased from Swapnroop Drugs&Pharmaceuticals (Aurangabad, India). Di‑sodium hydrogen phosphate dihydrate and sodium dihydrogen phosphate dihydrate for preparing the dissolution medium were obtained from Th. Geyer (Renningen, Germany).

### 2.2 Preparation of polymer placebo formulations and ASDs via vacuum compression molding (VCM)

Placebo formulations comprising EL 100–55: HPC-SSL (50:50 mass ratio) and EL 100–55: HPC-UL (50:50 mass ratio) were prepared via VCM. Binary ASDs with 10% drug load of EFV were processed, using EL 100–55, HPC-SSL, and HPC-UL as ASD-forming polymers. For determining the maximum kinetic solid-state solubility of EFV within the single polymers (3.6.), additional highly drug loaded binary ASDs (from 50% up to 70% drug load) were processed. Additionally, ternary VCM ASDs (10% drug load) with EL 100–55: HPC-SSL (50:50 mass ratio) and EL 100–55: HPC-UL (50:50 mass ratio) were prepared.

Prior to placebo and ASD processing, homogeneously milled physical mixtures (PMs) were prepared by blending the components in a MM400 ball mill (Retsch GmbH, Haan, Germany) for 15 min (3 × 5 min cycles) at a frequency of 30 Hz. For the binary and ternary ASDs, approx. 500 mg of the PMs were filled into the VCM device of 20 mm diameter disc geometry (MeltPrep GmbH, Graz, Austria) and heated at 160 °C for 15 min under vacuum, followed by cooling of approx. 10 min until the ASD discs were obtained. Prior to VCM processing, thermal stability of EFV and of the polymers for this processing conditions were investigated and confirmed via thermogravimetric analysis (data not shown).

For X-Ray powder diffraction (XRPD) (2.4.), differential scanning calorimetry (DSC) (2.5.), Fourier-transform infrared spectroscopy (FT-IR) (2.6), and the non-sink dissolution study (2.9) the VCM discs were milled using an MM400 ball mill (Retsch GmbH, Haan, Germany) with a frequency of 30 Hz. The obtained ASD particles were sieved (mesh size 355 μm) and larger fractions were excluded from further analysis.

### 2.3 Preparation of polymer placebo formulations and ASDs via hot-melt extrusion (HME)

Placebo formulations and ternary ASDs (10% drug load) using EL 100–55: HPC-SSL (50:50 mass ratio) and EL 100–55: HPC-UL (50:50 mass ratio) were prepared via HME.

The corresponding PMs were obtained using a Turbula® mixer (Willy A. Bachofen AG Maschinenfabrik, Switzerland), rotating at 50 rpm for 10 min. Subsequently, the PMs were fed with a constant rate of 2 g/min to a 12 mm co-rotating twin screw extruder ZE 12 (Three-Tec GmbH, Seon, Switzerland) with five heating zones (functional length of 25:1 L/D), a fixed screw configuration, and a 2 mm die. The division of the screw configuration into the conveying and kneading elements is provided in the Supplementary data (Fig. S1). The processing occurred, selecting a constant screw speed of 100 rpm, and setting the temperatures of the heating zones to 40/75/150/150/150 °C. Compared to the VCM process, the maximum processing temperature was slightly reduced, for generation of additional heat via viscous dissipation (increased shear forces within the kneading elements). The obtained extrudates were milled using a MM400 ball mill (Retsch GmbH, Haan, Germany) with a frequency of 30 Hz. Particles with a size >355 μm were excluded by sieving. To obtain a disc with uniform surface for the investigations of the HME placebo formulations via confocal Raman spectroscopy (CRS) (2.7), HME extrudate VCM discs were prepared, using the conditions of section 2.2.

### 2.4 X-Ray powder diffraction (XRPD)

XRPD was performed in reflection mode utilizing a X' Pert MRD Pro (PANalytical, Almelo, Netherlands) at 45 kV and 40 mA with a X'Celerator detector and nickel filtered CuKα1 radiation. Scans were conducted in a range from 5 to 45° 2θ and the step size was set to be 0.017° 2θ.

### 2.5 Differential scanning calorimetry (DSC)

To examine the phase behavior of the processed binary and ternary ASDs (10% drug load) and to investigate the maximum kinetic solid-state solubility of EFV within the single polymers with respect to the selected VCM processing conditions (160 °C for 15 min), DSC investigations were carried out by using a DSC 2 instrument (Mettler, Gießen, Germany). The device was equipped with nitrogen as purge gas and a nitrogen cooling system to enable a low starting temperature. Approx. 10 mg of the samples were weighed into an aluminum pan with a pierced lid. The melting point (onset) of neat EFV was investigated using a conventional method, consisting of a constant temperature rising of 10 °C/min from 0 to 170 °C. For determining the T_g_s (midpoint of the glass transition) and the potential melting points (onset) of the ASDs, the TOPEM-mode, a multi-frequency temperature-modulated program, was selected. The temperature programs were conducted with an underlying heat rate of 2 °C/min, starting from 0 °C and ending at 150 °C for the investigation of the EFV ASDs and ending at 170 °C for the placebo formulations. The investigations of the ASDs occurred immediately after the VCM process. All experiments were conducted in triplicates. The results of neat EL 100–55, of HPC-SSL, and of the EL 100–55: HPC-SSL placebo combinations were already collected and published in a previous investigation of our workgroup (Pöstges et al., 2022).

### 2.6 Fourier-transform infrared spectroscopy (FT-IR)

Solid-state interactions of the placebos and ASD formulations were analyzed via FT-IR, utilizing a Spectrum Two FT-IR spectrometer (PerkinElmer, Waltham, MA, USA). Sixteen scans of each sample were collected in a spectral range of 450–4000 cm^−1^. The spectra of neat EL 100–55, of HPC-SSL, and of the EL 100–55: HPC-SSL placebo combinations were already collected and published in a previous investigation of our workgroup (Pöstges et al., 2022).

### 2.7 Confocal Raman spectroscopy (CRS)

Distribution of the polymers and EFV in the VCM discs were investigated by CRS using an alpha 300R confocal Raman microscope (WiTec, Ulm, Germany), equipped with a 532 nm excitation laser, an UHTS 300 spectrometer, a DV401-BV CCD camera and a 40 × 0.6 NA objective. For the VCM samples and the neat polymers, the laser power was set to 25 mW. For neat EFV, the power was reduced to 10 mW. Characteristic shapes and peaks of the spectra of each raw material were selected for identifying the substances in the Raman image. The integration time was set to 2 s for the collection of the Raman spectra of the neat substances. For investigating the formulations, the integration time was decreased to 0.5 s. As a compromise between a sufficient sample size for investigating the phase behavior of the components and an appropriate measuring time, the scanning cutout of the VCM discs was selected to be 50 μm × 50 μm. Images were obtained by measuring 50 points per line and 50 lines per images, using the autofocus function of the microscope. Based on the intensities of the characteristic wavenumbers, individual color coded images for each component were created, revealing enhanced presence or absence of the corresponding substance in the formulations.

### 2.8 Melt rheology

The melt viscosity of the neat polymers EL 100–55, HPC-SSL, and HPC-UL were investigated, utilizing a small amplitude oscillatory rheometer (HAAKE MARS III, Thermo Scientific, Karlsruhe, Germany), equipped with a plate-plate geometry (d = 20 mm). VCM discs of neat polymers were obtained, using the processing conditions as described in section 2.2. and equilibrated for 10 min at 160 °C before performing the measurements. Subsequently, frequency sweeps (*n* = 3) were conducted from 10 Hz (62.83 rad/s) to 0.1 Hz (0.63 rad/s) at 160 °C. As the manufacturing technique VCM is a non-shear processing method, the complex viscosities at 1 rad/s were calculated to get an impression about the viscosities at minimal shear rate. The utilized amplitudes were determined to be in the linear viscoelastic range, prior.

### 2.9 Non-sink dissolution

Non-sink dissolution testing was performed by using a miniaturized USP dissolution apparatus II (MiniDissolution apparatus) (Zecevic and Wagner, 2013). To mimic intestinal pH conditions, a pH 6.8 phosphate buffer (0.05 M) was selected as dissolution medium. Stirrer speed of the paddles and the temperature of the dissolution medium were set to 75 rpm and 37 °C, respectively. A sample size of 40 mg ASD/ 4 mg neat API was added to 20 mL of the pre-warmed dissolution medium and the concentration of dissolved drug was measured for 180 min using an 8453 UV/VIS spectrophotometer (Agilent, Waldbronn, Germany), including correction for scattering.

## 3 Results

### 3.1 X-Ray powder diffraction (XRPD) of neat drug and ASDs (10% drug load)

**Fig. 1:**
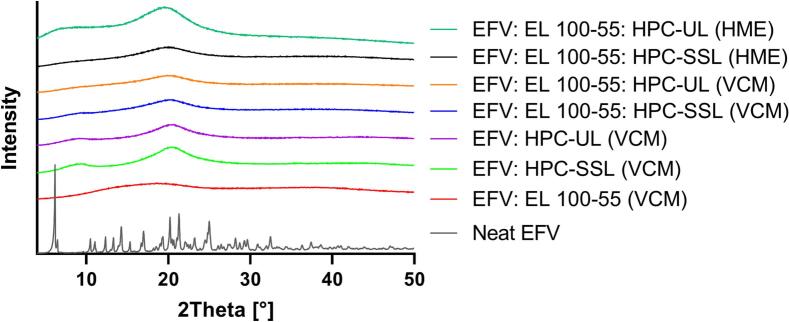
X-ray powder diffraction (XRPD) diffractograms: Neat EFV is presented with the processed ASD formulations (10% drug load).

In order to investigate potential residual crystallinity in the binary and ternary ASDs with 10% drug load XRPD measurements were performed (Fig. 1). Prior to the ASD investigations, the XRPD diffractogram of neat EFV was examined. Due to the natural crystalline structure of EFV, sharp reflection peaks of the neat drug were visible. However, for all investigated ASDs no reflection peaks were detected, indicating the complete amorphous character of embedded EFV.

### 3.2 Differential Scanning calorimetry (DSC) of neat polymers and ASDs (10% drug load)

**Fig. 2:**
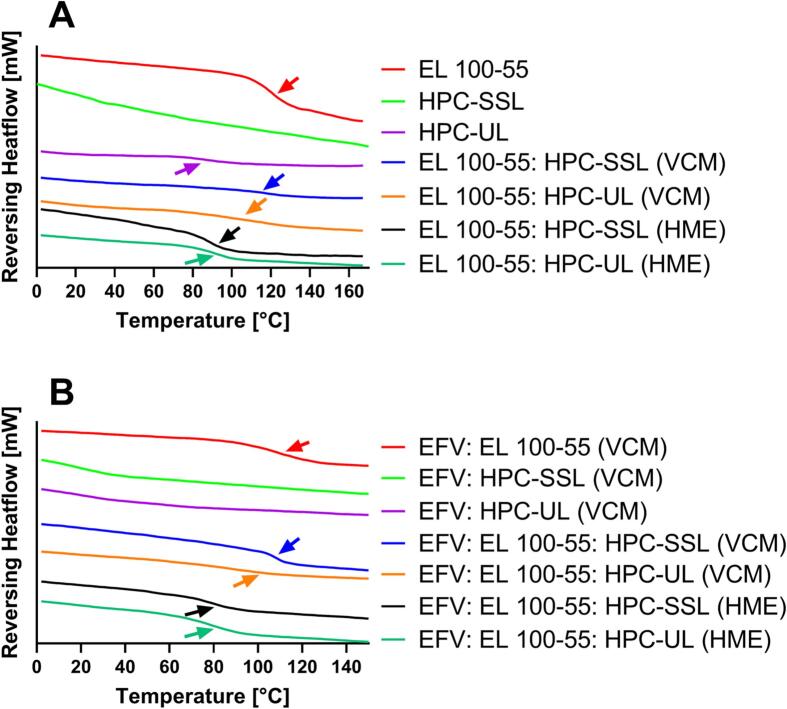
Differential scanning calorimetry (DSC) thermograms (exo up) of the neat polymers and placebo formulations (A), and of the EFV ASDs (10% drug load) (B).

An overview of the detected T_g_s of the single polymers, of the processed placebo formulations and of the EFV ASDs with 10% drug load are provided in Table 1 (T_g_ of neat EFV: 34 °C (Trasi et al., 2014)). The DSC thermograms of the single polymers and placebo formulations are depicted in Fig. 2A. The results of EL 100–55, HPC-SSL, and of the EL 100–55: HPC-SSL placebo formulations were already published in a previous study (Pöstges et al., 2022). For better understanding and for a complete data set, the data are presented, again.

**Table 1:** Glass transition temperatures (T_g_s) of all investigated placebo and ASD formulations.

Polymers	Preparation method		Glass transition temperatures (T_g_s) [°C]	
	VCM	HME	Placebo	EFV: ASD
EL 100–55	X		118.0 ± 0.1	107.3 ± 0.5
HPC-SSL	X		n/a	n/a
HPC-UL	X		81.1 ± 0.2	n/a
EL 100–55: HPC-SSL	X		117.8 ± 0.8	107.0 ± 0.5
EL 100–55: HPC-UL	X		106.1 ± 2.6	97.7 ± 1.2
EL 100–55: HPC-SSL		X	88.3 ± 0.3	79.2 ± 1.0
EL 100–55: HPC-UL		X	90.1 ± 0.6	79.6 ± 0.5

The T_g_ of EL 100–55 was determined to be 118.0 ± 0.1 °C. The glass transitions of the HPC polymers were only hardly detectable, as the step height of the reversing heat flows were very small for determining the T_g_s. However, in case of HPC-UL a T_g_ at 81.1 ± 0.2 °C was observed. This T_g_ corresponded very well to the previously reported HPC-UL T_g_ of Luebbert et al. (81.6 °C) who determined T_g_s of various HPC grades by extrapolating the glass transitions of spray-dried HPC: PVP-VA blends (Luebbert et al., 2020). As the T_g_ of HPC-SSL could not be determined, the study needs to refer to the extrapolated T_g_ of HPC-SSL (81.8 °C), published by Luebbert et al. The EL 100–55: HPC-SSL VCM and HME formulations showed manufacturing-dependent differences in the T_g_s, as for the VCM formulation the T_g_ of EL 100–55 was still detected, while the HME placebo demonstrated a new single T_g_ at 88.3 ± 0.3 °C. A similar T_g_ (90.1 ± 0.6 °C) was also observed for the EL 100–55: HPC-UL HME formulation. Interestingly, the EL 100–55: HPC-UL VCM placebo demonstrated neither the miscible T_g_ around 90 °C, nor the T_g_ of EL 100–55 around 118 °C. Instead, a broad temperature window of the glass transition was revealed and the T_g_ determined to be 106.1 ± 2.6 °C. For enhanced visualization of the differences in the glass transitions between EL 100–55: HPC-SSL VCM and EL 100–55: HPC-UL VCM, the corresponding DSC thermograms are presented rescaled, additionally (Fig. S2A in the Supplementary data).

Regarding the binary and ternary ASDs (Fig. 2B), a pronounced single T_g_ at 107.3 ± 0.5 °C was obtained for the EFV: EL 100–55 ASD, indicating the formation of a single-phased system. Due to too small step heights in the thermograms, T_g_s of the HPC ASDs could not be determined. In case of the EFV: EL 100–55: HPC-SSL VCM ASD, a T_g_ at 107.0 ± 0.5 °C was detected. As this T_g_ was very close to the T_g_ of the binary EFV: EL 100–55 ASD, the formation of a pronounced phase-separated ternary ASD was assumed. By processing the EFV: EL 100–55: HPC-SSL combination via HME, the T_g_ around 107 °C disappeared and a new single T_g_ at 79.2 ± 1.0 °C was observed.

The ternary HME ASD, consisting of HPC-UL instead of HPC-SSL, demonstrated comparable glass transition, as a single T_g_ at 79.6 ± 0.5 °C was determined, indicating the formation of a homogeneous ternary ASD. Similar to the corresponding placebo formulations the EFV: EL 100–55: HPC-UL VCM ASD demonstrated different solid-state behavior compared to the ternary HME ASDs and to the EFV: EL 100–55: HPC-SSL VCM ASD. As the T_g_ was determined to be 97.7 ± 1.2 °C, neither a pronounced phase-separation, nor a formation of a complete homogenous system was assumed. The rescaled presentation of the DSC thermograms of the ternary VCM ASDs underlines the different phase behavior in dependence on the selected HPC grade (Fig. S2B of the Supplementary data).

### 3.3 Fourier-transform infrared spectroscopy (FT-IR)

**Fig. 3:**
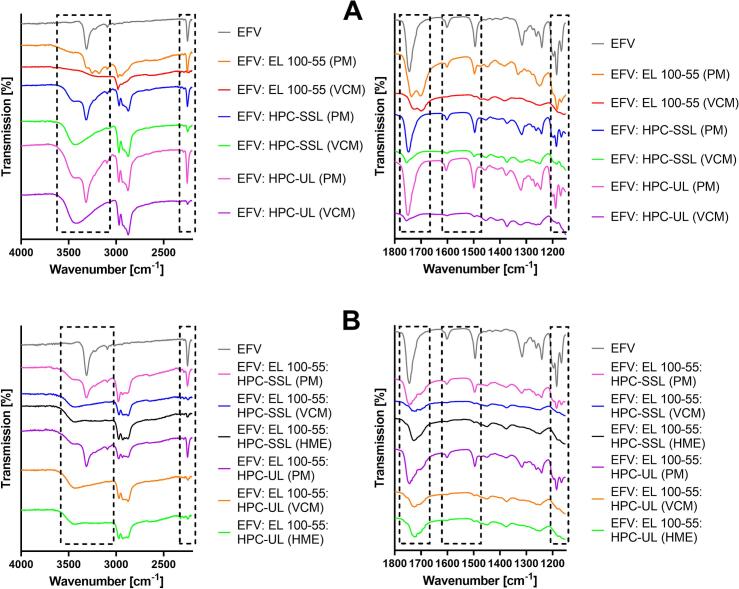
Fourier-transform infrared (FT-IR) spectra of the binary (A) and ternary (B) EFV formulations compared to neat EFV and the corresponding physical mixtures (PMs).

Fig. 3 depicts the relevant FT-IR spectra ranges of neat EFV compared to the binary PMs/ ASDs (Fig. 3A) and to the ternary PMs/ ASDs (Fig. 3B). For enhanced visualization of the data the spectra are split, presenting the spectral range from 2200 cm^−1^ to 4000 cm^−1^ on the left side and the range from 1150 cm^−1^ to 1800 cm^−1^ on the right side of the figure. The FT-IR spectra of the neat polymer(s) and of the placebo formulations are provided in the Supplementary data (Fig. S3).

The measurement of neat EFV showed NH stretching vibrations (3311 cm^−1^), C≡C stretching vibrations (2249 cm^−1^), C=O vibrations (1744 cm^−1^), C=C vibrations of the benzene ring (1600 cm^−1^ and 1494 cm^−1^), and C—O vibrations (1184 cm^−1^). All described peaks were visible in the investigated PMs. Regarding the PM of EFV: EL 100–55 in Fig. 3A, the double peak at 1701 cm^−1^ and 1736 cm^−1^, representing additional C=O stretching vibrations of the carboxylic acid groups and of the esterified carboxyl groups of EL 100–55, respectively, were detected. In addition to the EFV peaks, the binary PMs, consisting of HPC-SSL or HPC-UL, demonstrated a broad OH peak around 3400-cm^−1^.

Regarding the FT-IR spectra of the binary ASD (Fig. 3A), both the EL 100–55 and the HPC ASDs demonstrated interactions with NH and C—O of EFV, as the corresponding peaks vanished completely. For the binary HPC ASDs peak shifts from 1747 cm^−1^ (PMs) to 1755 cm^−1^ indicated additional interactions including the C=O of EFV. Furthermore, the C≡C and C=C vibrations of the benzene ring of EFV lost intensity after processing with EL 100–55, indicating further interactions between drug and polymer.

The FT-IR spectra of the processed ternary ASDs (Fig. 3B) demonstrated comparable solid-state interactions with EFV, as again the stretching vibrations of NH and C—O of processed EFV were not detected and the shape of the C=O vibrations was affected. However, regarding the FT-IR bands of the polymers, differences between the VCM and the HME processed ternary ASDs were observed. Both HME ASDs demonstrated stronger decrease of the OH bands of the HPC polymers compared to the corresponding VCM formulations. In addition, the EL 100–55: HPC HME formulations showed shape changes of the C=O double peaks, as the peaks at 1701 cm^−1^ reduced in intensity, indicating interactions between the carboxylic acid groups of EL 100–55 with the HPC polymers. These polymer-polymer interactions were also visible in the placebo formulations, depicted in the Supplementary data (Fig. S3). Additional manufacturing-dependent drug-polymer interactions in the ternary ASDs could not be detected.

### 3.4 Confocal Raman spectroscopy (CRS)

#### 3.4.1 Placebo formulations

**Fig. 4:**
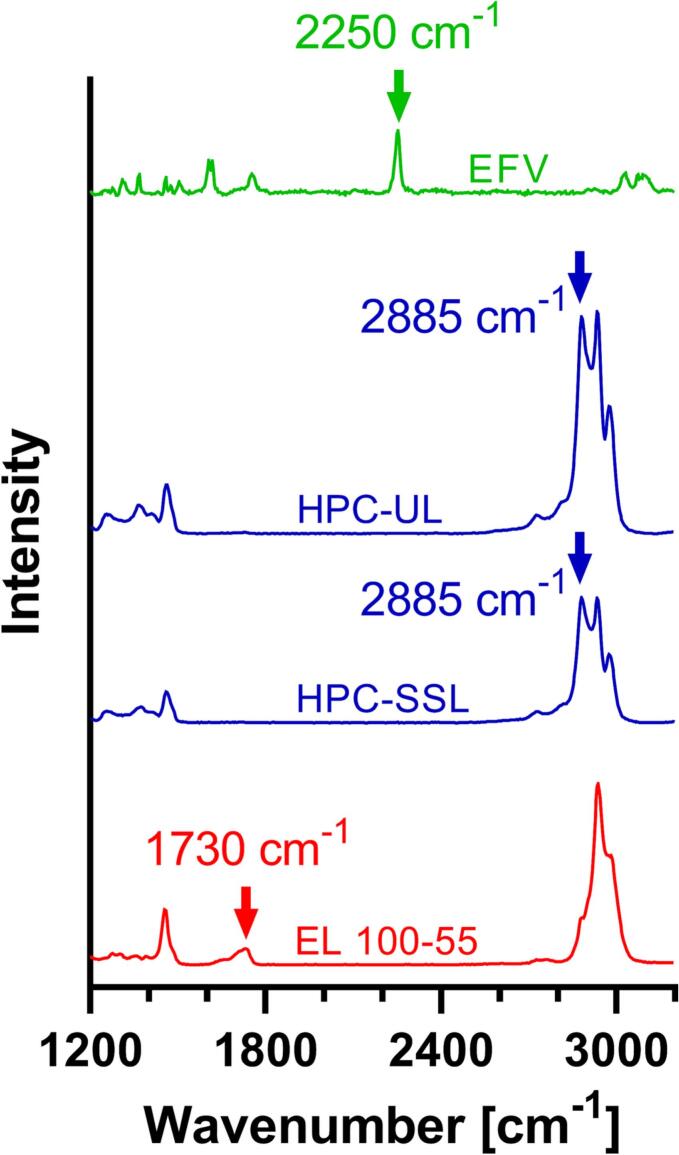
Single Raman spectra of the neat components for the investigation of the corresponding placebos and ASD formulations. The characteristic wavenumbers for identifying the components within the formulations are marked with arrows.

The solid-state of the polymer placebo formulations were characterized via CRS. The single Raman spectra of the neat polymer revealed differences in peak shapes and intensities (Fig. 4). EL 100–55 showed a characteristic peak at 1730 cm^−1^ (red arrow) while in the spectra of the HPC polymers no Raman signal was observed at this wavenumber. Therefore, the Raman intensity at 1730 cm^−1^ was used for detecting EL 100–55. In contrast to EL 100–55, the HPC polymers revealed a characteristic double peak at 2885 cm^−1^ and 2937 cm^−1^. The intensity of the front peak at 2885 cm^−1^ (blue arrow) was suitable for identifying enhanced presence of the HPC polymers.

**Fig. 5:**
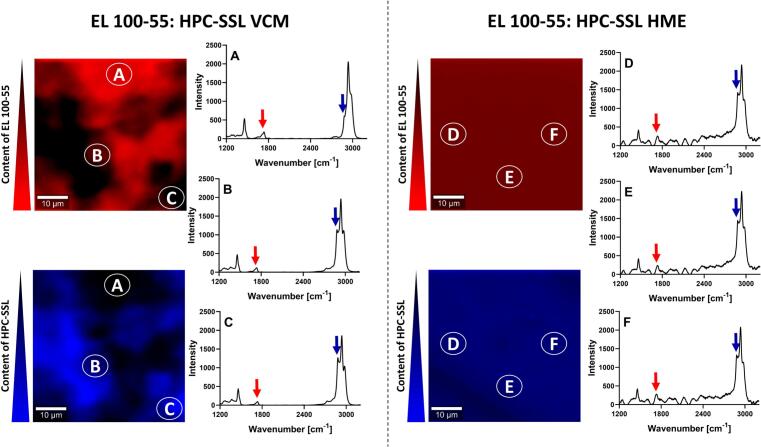
Confocal Raman spectroscopy (CRS) of EL 100–55: HPC-SSL placebo formulations, processed by vacuum compression molding (VCM) (A, B, C) and by hot-melt extrusion (HME) (D, E, F). In the upper images, the intensity at 1730 cm^−1^ was utilized for detecting the presence of EL 100–55 (red color). In the lower images, the intensity at 2885 cm^−1^ was used to identify the presence of HPC-SSL (blue color). (For interpretation of the references to color in this figure legend, the reader is referred to the web version of this article.)

Fig. 5 demonstrates arbitrary selected investigated areas of the EL 100–55: HPC-SSL formulations. The images on the left represent the formulation processed by VCM, the images on the right represent the HME extrudate. In the corresponding upper images, the intensity of the peak at 1730 cm^−1^ was used for identifying EL 100–55. The color red demonstrates high Raman intensity at 1730 cm^−1^ and thus, the presence of EL 100–55. Instead, in the lower images the blue color is a result of high intensity at 2885 cm^−1^, revealing the presence of HPC-SSL.

For the EL 100–55: HPC-SSL VCM formulation, different Raman spectra were detected, indicating a heterogeneous mixture. High content of EL 100–55, depicted by intensive red color, was detected when the Raman intensity for identifying HPC-SSL was very low (Fig. 5A). The corresponding Raman spectrum revealed similar spectral information compared to the collected spectrum of neat EL 100–55, indicating distinct phase-separation of both polymers. Instead, other spots of the investigated areas showed reduced presence of EL 100–55, but higher content of HPC-SSL, as the blue color intensified (Fig. 5B and C).

For the EL 100–55: HPC-SSL HME formulation, consistent intensities at 1730 cm^−1^ and at 2885 cm^−1^ were observed, represented by the same red and blue color for the entire investigated section. The Raman spectra of arbitrary selected investigated areas in Fig. 5 are marked with the letter D, E, and F, respectively. All three Raman spectra demonstrated comparable peak intensities and peak shapes, indicating a homogeneous polymer mixture.

**Fig. 6:**
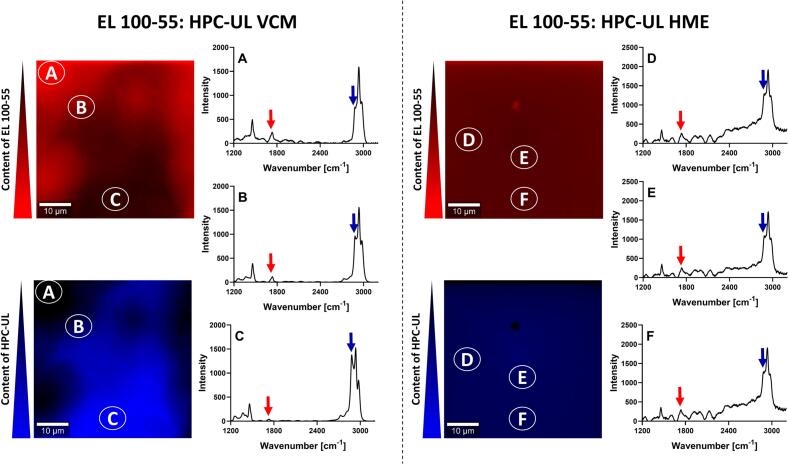
Confocal Raman spectroscopy (CRS) of EL 100–55: HPC-UL placebo formulations, processed by vacuum compression molding (VCM) (A, B, C) and by hot-melt extrusion (HME) (D, E, F). In the upper images, the intensity at 1730 cm^−1^ was utilized for detecting the presence of EL 100–55 (red color). In the lower images, the intensity at 2885 cm^−1^ was used to identify the presence of HPC-UL (blue color). (For interpretation of the references to color in this figure legend, the reader is referred to the web version of this article.)

Fig. 6 demonstrates the CRS images of the EL 100–55: HPC-UL placebo formulations. The observations were comparable to the EL 100–55: HPC-SSL placebos. The VCM disc revealed local differences in Raman intensity at 1730 cm^−1^ and 2885 cm^−1^, leading to inhomogeneous color distributions as result of heterogeneous polymer phases. Both colors demonstrated complementary appearances, thus higher presence of EL 100–55 was associated with lower presence of HPC-UL and vice versa. However, the HME processed polymer mixture of EL 100–55 and HPC-UL showed comparable Raman signals within the entire investigated section, indicating a single-phased polymer mixture.

#### 3.4.2 Ternary ASD formulations (VCM)

For investigating the ternary ASD formulations (VCM) the single Raman spectrum of neat EFV was determined, additionally. EFV revealed a characteristic Raman shift at 2250 cm^−1^, hence the corresponding intensity was used for identifying EFV in the polymer matrix, indicated by the green arrow in Fig. 4. The intensities at 1730 cm^−1^ and 2885 cm^−1^ were still suitable for detecting EL 100–55 (red arrow) and HPC (blue arrow), respectively.

**Fig. 7:**
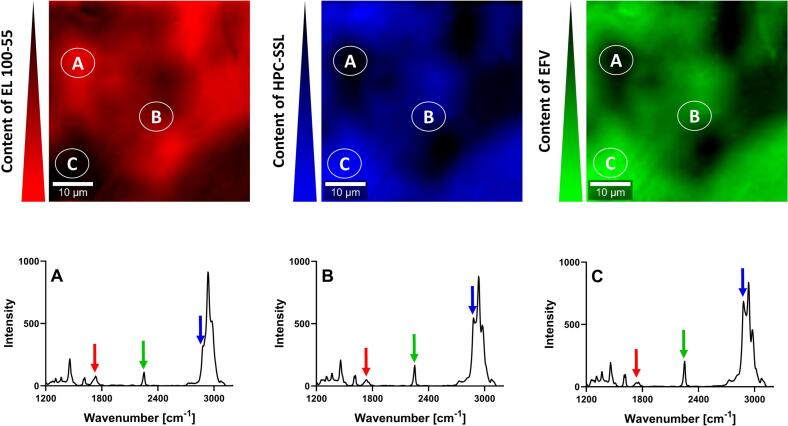
Confocal Raman spectroscopy (CRS) of the EFV: EL 100–55: HPC-SSL ASD (VCM), using the intensity of 1730 cm^−1^ and 2885 cm^−1^ for detecting EL 100–55 (red color) and HPC-SSL (blue color), respectively. The intensity at 2250 cm^−1^ revealed the distribution of EFV (green color). (For interpretation of the references to color in this figure legend, the reader is referred to the web version of this article.)

Fig. 7 represents the EFV: EL 100–55: HPC-SSL (VCM) ternary ASD. For the green-colored image, the intensity at 2250 cm^−1^ was utilized for visualizing the presence of EFV. Therefore, high intensity of the green color indicates high content of EFV, while the dark color represents low content of EFV. Similar to the investigations of the placebo formulations (section 3.4.1), the red-colored and blue-colored images reveal the presence of EL 100–55, and HPC, respectively.

The inconsistent green color distribution as a result of the heterogeneous Raman intensities at 2250 cm^−1^ indicated inconsistent distribution of EFV within the ternary ASD. Noticeable, the distribution of the colors for revealing the distribution of EFV and HPC-SSL were very comparable. Instead, the green color for EFV and the red color for EL 100–55 showed complementary appearances. Exemplary, Fig. 7 spot A demonstrated a local area with low content of EFV and HPC-SSL, but high content of EL 100–55. Instead, Fig. 7 spot C revealed a local area with high amount of EFV and HPC-SSL, while only little intensity of the characteristic peak of EL 100–55 (1730 cm^−1^) was detected. Apparently, during the VCM melting process, EFV distributed preferably into the HPC-SSL richer phases than into the EL 100–55-richer phases. Additionally, moderate content of EFV was detected in areas, represented at Fig. 7 spot B.

**Fig. 8:**
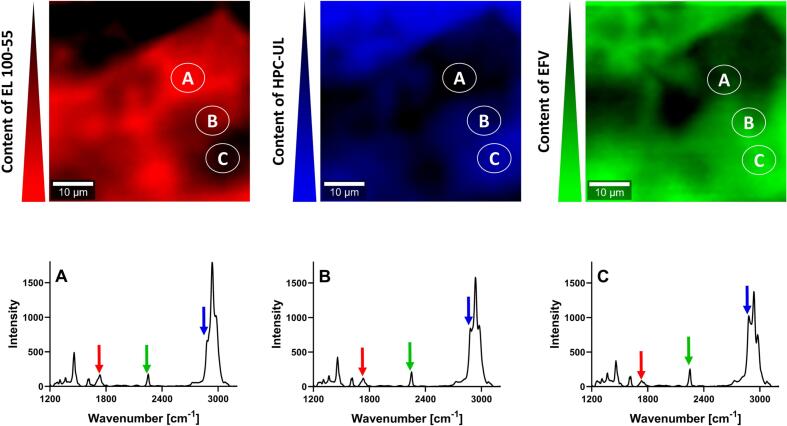
Confocal Raman spectroscopy (CRS) of the EFV: EL 100–55: HPC-UL ASD (VCM), using the intensity of 1730 cm^−1^ and 2885 cm^−1^ for detecting EL 100–55 (red color) and HPC-UL (blue color), respectively. The intensity at 2250 cm^−1^ revealed the distribution of EFV (green color). (For interpretation of the references to color in this figure legend, the reader is referred to the web version of this article.)

Regarding the EFV: EL 100–55: HPC-UL ASD (VCM) similar results were obtained, as inhomogeneous distribution for both EFV and the polymers was demonstrated (Fig. 8). The color distributions and the representing Raman spectra at spots A, B, and C showed that higher Raman intensity at 2250 cm^−1^ (for detecting EFV) was associated with lower Raman intensity at 1730 cm^−1^ (for detecting EL 100–55) and vice versa. Instead, high content of EFV was detected in HPC-UL-richer phases, as the green-colored and blue-colored image demonstrated comparable color distribution.

### 3.5 Melt viscosity of neat polymers

**Fig. 9:**
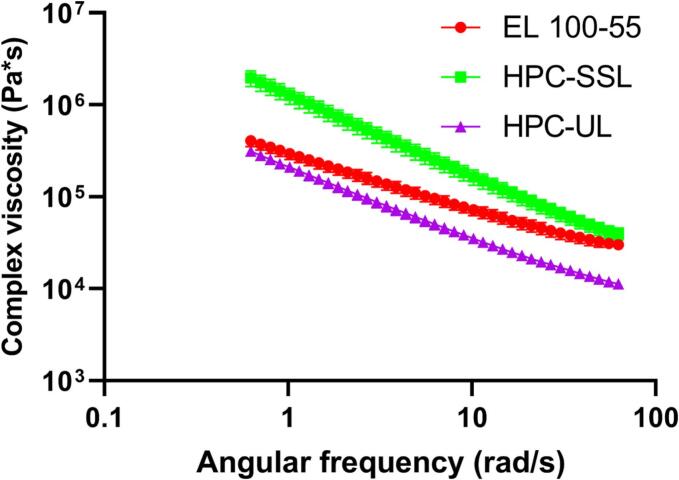
Frequency sweeps of the neat polymers EL 100–55, HPC-SSL, and HPC-UL at the VCM processing temperature of 160 °C.

To find a potential explanation for the observations of the confocal Raman microscope, the melt viscosities of the neat polymers were investigated via frequency sweeps (Fig. 9) and the complex viscosities at minimal shear rate (1 rad/s) were calculated for a temperature of 160 °C. All polymers demonstrated shear-thinning behavior, as the viscosities were increased by reducing the angular frequency. Regarding high frequencies EL 100–55 and HPC-SSL exhibited comparable complex viscosities. However, by reducing the shear rate the complex viscosities differed decisively, leading to calculated viscosities of 299,406 Pa*s and 1,287,189 Pa*s for EL 100–55 and HPC-SSL, respectively, at 1 rad/s (Table 2). Compared to HPC-SSL, HPC-UL showed comparable shear-thinning behavior. However, the complex viscosities were significantly reduced, as HPC-UL revealed an extrapolated viscosity of 213,085 Pa*s at 1 rad/s.

**Table 2:** Calculated complex viscosities (Pa*s) at an angular frequency of 1 rad/s for the neat polymers EL 100–55, HPC-SSL, and HPC-UL at the VCM processing temperature of 160 °C.

Polymer	Complex viscosity [Pa*s] at 1 rad/s
EL 100–55	299,406 ± 41,635
HPC-SSL	1,287,189 ± 252,153
HPC-UL	213,085 ± 23,830

### 3.6 Differential Scanning calorimetry (DSC) of highly drug loaded binary ASDs

**Fig. 10:**
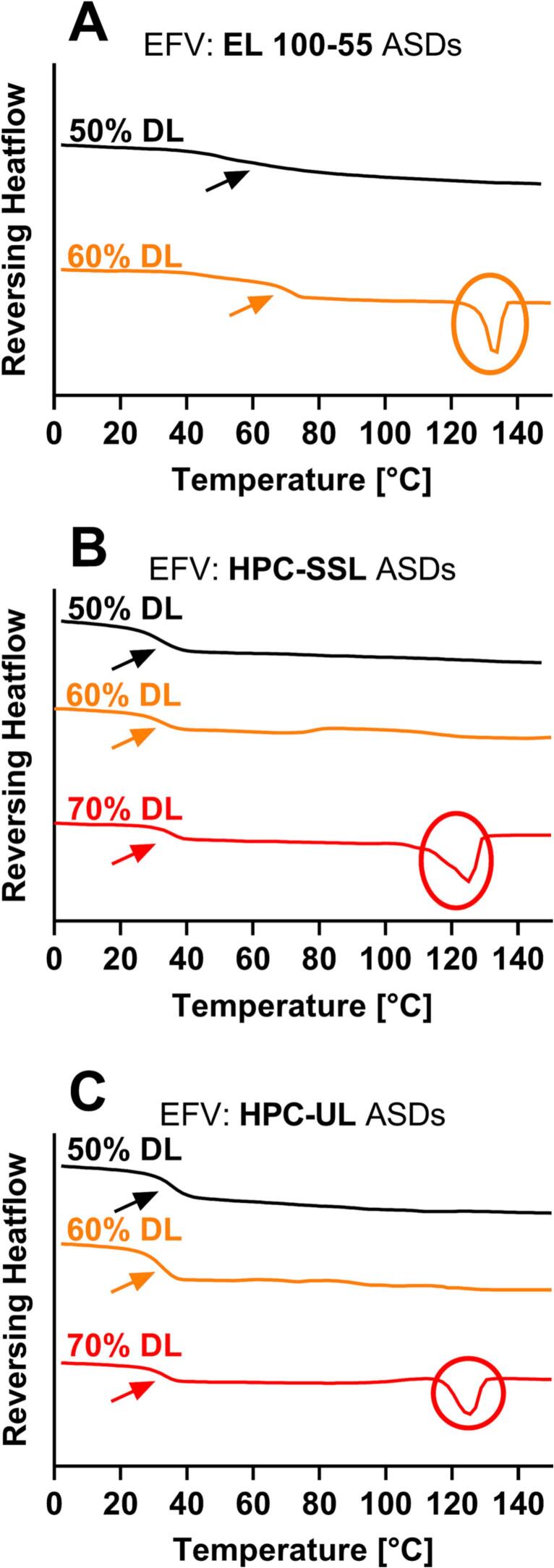
Differential scanning calorimetry (DSC) thermograms (exo up) of highly drug loaded binary EFV ASDs, using EL 100–55 (A), HPC-SSL (B), and HPC-UL (C) as ASD-forming polymers, respectively.

The maximum kinetic solid-state solubility of EFV in EL 100–55 and in the HPC polymers (binary ASDs) with respect to the selected VCM processing conditions (160 °C for 15 min) were investigated via DSC (Fig. 10). Single T_g_s and the absence of a melting peak would indicate complete miscibility, while the detection of residual crystallinity would indicate that the kinetic solubility was exceeded at 160 °*C.* Prior to the ASD investigations, the melting point of EFV was determined to 137.8 ± 0.1 °C. The corresponding DSC thermogram can be found in the Supplementary data (Fig. S4). All binary ASDs with a drug load of 50% demonstrated single T_g_s and the absence of the EFV melting point (Fig. 10). By increasing the drug load to 60%, the EFV: EL 100–55 showed a pronounced melting peak at 128.9 °C (Fig. 10A), while in case of both HPC polymers EFV was embedded completely amorphous and single-phased into the polymer matrix (Fig. 10B and C). By further increasing to 70% drug load, both the HPC-SSL and the HPC-UL ASDs revealed a depressed melting point of EFV at 117.3 and 117.8 °C, respectively.

### 3.7 Non-sink dissolution study

**Fig. 11:**
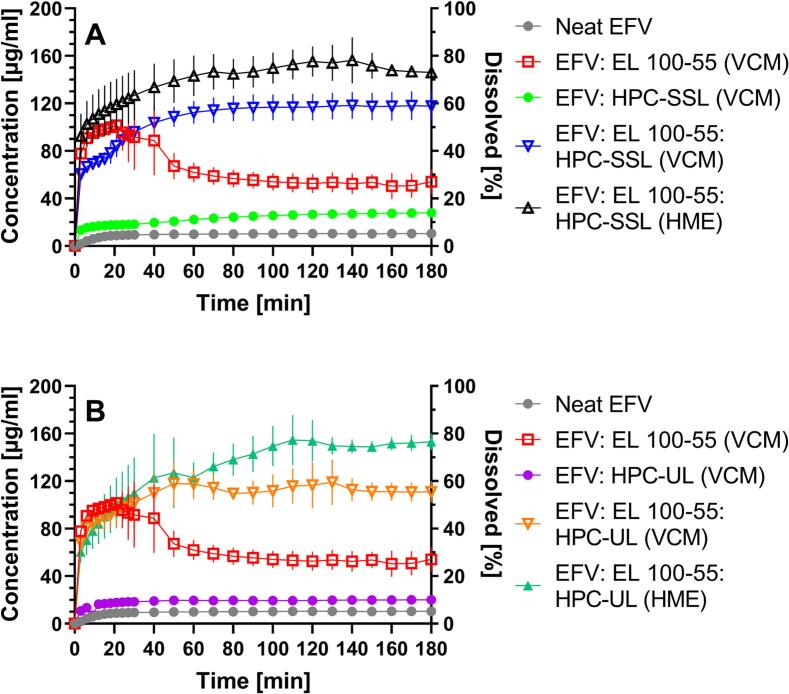
Non-sink dissolution study of the ASD formulations (10% drug load), comprising EFV, EL 100–55, HPC-SSL (A), and EFV, EL 100–55, HPC-UL (B), compared to neat EFV. The dissolution testing was performed in 20 mL 0.05 M phosphate buffer at pH 6.8 (37 °C, 75 rpm paddle speed). For enabling high non-sink conditions, the samples size corresponded to 4 mg EFV (=40 mg formulation), resulting in max. concentrations of 0.2 mg/mL dissolved EFV (=100%).

Fig. 11 demonstrates the non-sink dissolution results of the ternary EFV ASDs, processed via VCM and HME, compared to the corresponding binary ASD formulations and the neat drug. Fig. 11A presents the EFV dissolutions using the polymers EL 100–55 and HPC-SSL and Fig. 11B shows the results with the polymers EL 100–55 and HPC-UL, instead. Considerable manufacturing-dependent differences in particle size distributions within the EFV: EL 100–55: HPC-SSL ASDs and the EFV: EL 100–55: HPC-UL ASDs that could affect the dissolution rate were excluded prior to dissolution (Fig. S5 of the Supplementary data).The poor solubility of crystalline unprocessed EFV was clearly visible, as a maximal solubility of 10.4 μg/mL was detected. Embedding the drug into EL 100–55 led to a high initial dissolution rate and to a concentration of 90.7 μg/mL of dissolved EFV after 6 min. However, c_max_ (101.6 μg/mL) was obtained after 21 min, as the concentration started to decrease, leading to a final concentration of 54.0 μg/mL after the end of the observation period. Instead, the HPC-SSL ASD demonstrated low impact on the solubility enhancement, but a slightly increasing EFV concentration to 27.8 μg/mL after 180 min. Dissolving the VCM processed ternary EFV: EL 100–55: HPC-SSL ASD, the initial concentration of dissolved EFV (66.8 μg/mL after 6 min) was lower compared to the binary EL 100–55 ASD. However, the concentration increased constantly, leading to a concentration of approx. 116 μg/mL after 70 min that was maintained for the entire dissolution testing. The EFV: EL 100–55: HPC-SSL HME ASD demonstrated superior dissolution performance compared to the ternary VCM ASD, as the HME ASD revealed the fastest dissolution rate and max. EFV concentration of 156.2 μg/mL after 140 min. This concentration was stabilized for the entire dissolution with only small decrease in concentration, leading to a final concentration of 146.1 μg/mL after 180 min.

Comparable to the binary EFV: HPC-SSL ASD, the EFV: HPC-UL ASD showed low impact on solubility enhancement of EFV, leading to a final concentration of 20.0 μg/mL after 180 min (Fig. 11B). However, both the ternary VCM and the ternary HME ASD depicted fast dissolution rate and a stabilization of the supersaturated state for the entire observation period. Noticeably, the dissolution rates of the ternary EFV: EL 100–55: HPC-UL VCM ASD and of the corresponding ternary HME ASD were comparable for the first 60 min, followed by superior dissolution performance of the HME ASD. While the VCM ASD ended in a final concentration of 111.0 μg/mL, a concentration of 153.1 μg/mL was obtained at the end of the HME ASD dissolution.

## 4 Discussion

The dissolution testing of the ternary EFV ASDs revealed superiority in terms of solubility enhancement and supersaturation stabilization compared to the corresponding binary ASDs. The selected manufacturing technique (VCM or HME) and the selected HPC grade (HPC-SSL or HPC-UL) influenced the dissolution performance of the ternary ASDs.

Analyzing the solid-state of the ternary ASDs in detail provided insights into the mechanism upon dissolution and explanations for the differences between the dissolutions. The DSC thermograms and the CRS revealed differences of polymer mixing in dependence on the selected manufacturing method and HPC grade. Limitations in detecting phase homogeneity need to be considered, as the spatial resolution of our CRS method was in micro-meter range (approx. 3 μm), and DSC is not able to detect phase-separated domains smaller than 30 nm (Duan et al., 2020; Krause and Iskandar, 1977). Consequently, influence of potential smaller nanosized phase-separated domains on dissolution were not considered.

In a previous investigation of our workgroup, the manufacturing-dependent polymer mixing of EL 100–55 and the HPC grade -SSL using CXB as model drug was already detected via DSC. However, further characterizations of the inhomogeneous polymer blend (immiscible or partially miscible, and drug distribution) were not conducted (Pöstges et al., 2022).

By performing CRS, this current study provides deeper insights in terms of the phase behavior of the polymers and drug. Comparable Raman spectra within the whole investigated area of the HME placebo formulations and single T_g_s of the ternary HME ASDs indicated single-phased polymer mixtures and homogeneously embedding of EFV within the polymer matrices. By processing the same PMs via VCM, inhomogeneous distributions of the polymers and of EFV were detected, as heterogeneous polymer phases with either higher content of EL 100–55 or HPC were identified. In dependence on phase homogeneity, different extent of polymer-polymer interactions were detected, as the single-phased formulations demonstrated stronger intermolecular polymer interactions than the heterogeneous formulations, while the drug-polymer interactions did not seem to be impacted by the manufacturing method. Interestingly, although CRS was not able to depict differences between the VCM processed polymers, the VCM placebo consisting of HPC-UL revealed different phase behavior compared to the VCM processed EL 100–55: HPC-SSL formulation. While the EL 100–55: HPC-SSL VCM demonstrated a similar T_g_ compared to neat EL 100–55, the detected T_g_ of the EL 100–55: HPC-UL VCM shifted towards the T_g_ of the homogeneously mixed HME placebos. As partially miscible phases are characterized by new T_g_s, close to the ones of the unprocessed polymers (Lyu et al., 2005), the DSC investigations of the VCM processed formulations indicated higher content of miscible phases between EL 100–55 and HPC-UL compared to EL 100–55 and HPC-SSL. Potentially, due to the decisive smaller melt viscosity of HPC-UL compared to HPC-SSL, local homogeneous polymer phases with EL 100–55 in the VCM formulations were formed easier. The lower molecular weight of HPC-UL (20,000 g/mol) was likely the reason for the lower melt viscosity compared to HPC-SSL (40,000 g/mol), as it is well-known that the melt viscosity of one polymer group increases with increasing molecular weight (Alsulays et al., 2015; Shi et al., 2004; Thumma et al., 2008). However, to enable a homogeneous system, even for the EL 100–55: HPC-UL combination, shear forces generated by the kneading elements in HME were necessary. As in case of immiscible/ partially miscible polymer blends, investigation of the drug distribution is important to understand dissolution phenomena, the localization of EFV was further analyzed. As higher Raman signals of EFV were associated with higher Raman signals of the HPC polymers and lower signals of EL 100–55, EFV distributed preferably into the HPC-SSL- or HPC-UL-richer phases than in EL 100–55 during the melting processes of the ternary VCM ASDs. Diffusion based drug distribution would require lower complex viscosities of HPC polymers compared to EL 100–55. This was shown for HPC-UL, however, as HPC-SSL demonstrated significantly higher melt viscosity at minimal shear rate (1 rad/s) compared to EL 100–55, the diffusion could not be the driving factor for drug distribution. Instead, the drug distribution based mainly on the higher kinetic solid-state solubility of EFV within the HPC polymers than in EL 100–55. Comparable observations were published by Yang et al. where inconsistent drug distribution of a phase-separated ternary felodipine PVP-VA: Eudragit® E PO (50:50 polymer blend) ASD was described. The authors detected higher content of felodipine in the polymer (PVP-VA) in which the drug also demonstrated the higher solid-state solubility (Yang et al., 2013).

Comparable to the previous study with CXB (Pöstges et al., 2022), a homogeneous and intimate EL 100–55: HPC-SSL polymer mixture was required for enabling the full potential of the synergistic interactions and to achieve the maximum of EFV supersaturation. EL 100–55 alone showed high ability in increasing the EFV solubility rapidly, but less potential in maintaining the supersaturated state. Instead, HPC-SSL alone acted as an excellent precipitation inhibitor, but had only poor impact on the initial solubility of EFV. As EFV distributed preferably into the HPC-richer phases of the ternary VCM ASDs, especially the initial generation of the EFV supersaturation upon dissolution was decreased compared to the dissolution of the homogeneous ternary HME ASD. Since it is well-known that drug-polymer interactions influence dissolution (Hiew et al., 2022; Que et al., 2019), but FT-IR did not indicate manufacturing-dependent differences in the extent of drug-polymer interactions, the phase-separated polymer domains with lower polymer-polymer interactions were apparently the main factor for the reduced supersaturation of VCM processed EFV.

Similar to HPC-SSL, HPC-UL alone was not suitable as ASD-forming polymer, as the initial EFV solubility was only slightly improved compared to the dissolution of neat EFV. However, the dissolution performances of the ternary EFV: EL 100–55: HPC-UL ASDs were less dependent on the preparation technique compared to the ternary EFV: EL 100–55: HPC-SSL ASDs. Especially the initial dissolutions of the ternary VCM and HME ASDs were very comparable and the superiority of the HME ASD became only visible after 60 min of the dissolutions. Apparently, the higher content of miscible polymer phases within the heterogeneous ternary EFV: EL 100–55: HPC-UL VCM ASD was sufficient for executing comparable synergistic polymer interactions upon dissolution, compared to the corresponding HME ASD.

## 5 Conclusion

Polymer mixing of EL 100–55 and HPC was demonstrated to be dependent on the processing technique (VCM or HME) and selection of the utilized HPC grade (-SSL or -UL). Single-phased and homogeneous ternary ASDs were only prepared via HME. Instead, due to the missing shear forces partially miscible and heterogeneous ternary ASDs with higher EFV distribution within the HPC-rich phases were obtained via VCM. However, due to the lower melt viscosity of HPC-UL compared to HPC-SSL higher content of partially miscible phases with EL 100–55 were formed during VCM. As miscible phases were demonstrated to be important for the maximum enhancement of the dissolution of EFV, the ternary HME ASD consisting of HPC-SSL outperformed the corresponding VCM ASD, while only a small difference between the HME and VCM ASD, consisting of HPC-UL, was observed. Thus, especially regarding shear sensitive drugs, the use of HPC-UL is favorable, as higher polymer mixing with EL 100–55 using lower shear rates can be obtained.

## Funding

This work was funded and supported by Nippon Soda CO., Ltd., Tokyo, Japan. University of Bonn, University of Tübingen, and Nisso Chemical Europe GmbH participated in study design, research, interpretation of data, writing, data collection, analysis, reviewing, and approving the publication. Edmont Stoyanov is an employee of Nisso Chemical Europe GmbH and does not own stocks on Nippon Soda Ltd. Florian Pöstges is a PhD student and Karl G. Wagner a professor at the department of Pharmaceutical Technology and Biopharmaceutics at the University of Bonn, Germany. Jonas Lenhart is a PhD student and Dominique J. Lunter a professor at the department of Pharmaceutical Technology at the University of Tübingen, Germany. They have no additional conflicts of interest to report.

## CRediT authorship contribution statement

**Florian Pöstges:** Conceptualization, Methodology, Investigation, Data curation, Writing – original draft, Writing – review & editing, Visualization. **Jonas Lenhart:** Methodology, Investigation, Writing – review & editing. **Edmont Stoyanov:** Conceptualization, Resources, Writing – review & editing. **Dominique J. Lunter:** Writing – review & editing, Supervision. **Karl G. Wagner:** Conceptualization, Methodology, Resources, Writing – review & editing, Supervision.

## Declaration of Competing Interest

The authors declare the following financial interests/personal relationships which may be considered as potential competing interests:

Karl G. Wagner reports financial support was provided by Nippon Soda Co Ltd. Edmont Stoyanov reports a relationship with Nisso Chemical Europe Gmbh that includes: employment. Nisso Chemical Europe GmbH participated in study design, research, interpretation of data, analysis and reviewing the publication. The other authors do not have additional conflicts of interest to report.

## Data Availability

Data will be made available on request.

## Appendix A Supplementary data

Supplementary material Image 1
