# Supplementary material for: Phase homogeneity in ternary amorphous solid dispersions and its impact on solubility, dissolution and supersaturation – Influence of processing and hydroxypropyl cellulose grade
Source: Int J Pharm X. 2023 Dec 2;6:100222. doi: 10.1016/j.ijpx.2023.100222 (PMC10755049; doi:10.1016/j.ijpx.2023.100222)
Supplement: Supplementary file 1 — Supplementary material [file mmc1.pdf]

Supplementary Materials:

Phase homogeneity in ternary amorphous solid dispersions and its impact on solubility, dissolution and supersaturation – Influence of processing and hydroxypropyl cellulose grade

Florian Pöstges [a], Jonas Lenhart [b], Edmont Stoyanov [c], Dominique J. Lunter [b], Karl G. Wagner [a, *]

[a] *Department of Pharmaceutical Technology and Biopharmaceutics, University of Bonn, Gerhard-Domagk-Str. 3, 53121 Bonn, Germany*

[b] *Department of Pharmaceutical Technology, Faculty of Sciences, University of Tübingen, Auf d. Morgenstelle 8, 72076 Tübingen, Germany*

[c] *Nisso Chemical Europe GmbH, Berliner Allee 42, 40212 Düsseldorf, Germany*

* Corresponding author: karl.wagner@uni-bonn.de (Karl G. Wagner)

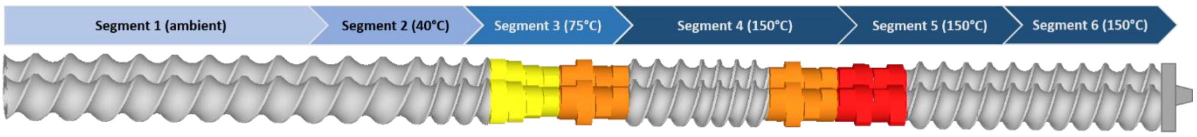

Fig. S1: Fixed screw configuration of the extruder for preparing the HME placebo formulations and the ternary EFV HME ASDs. The conveying elements are shown in grey color (9, 12, and 18 mm pitch). Depending on the staggering angle, the kneading elements are presented in yellow color (30°), orange color (60°), and in red color (90°). The temperatures of the segments were selected to be of ambient temperature (segment 1), 40 °C (segment 2), 75 °C (segment 3), 150 °C (segment 4, 5 and 6).

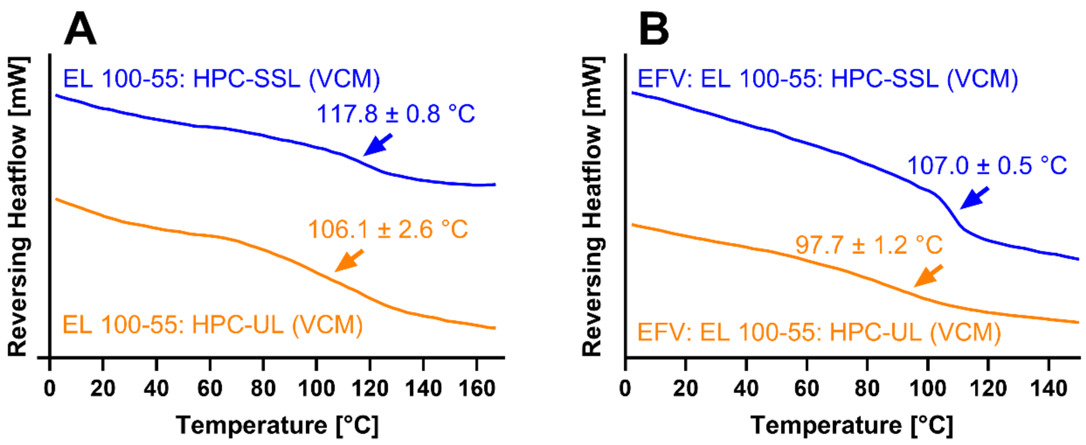

Fig. S2: Rescaled differential scanning calorimetry (DSC) thermograms of the vacuum compression molded (VCM) placebo formulations (A) and VCM processed ternary ASDs (B) for enhanced visualization of differences in phase behavior in dependence on the selected HPC grade.

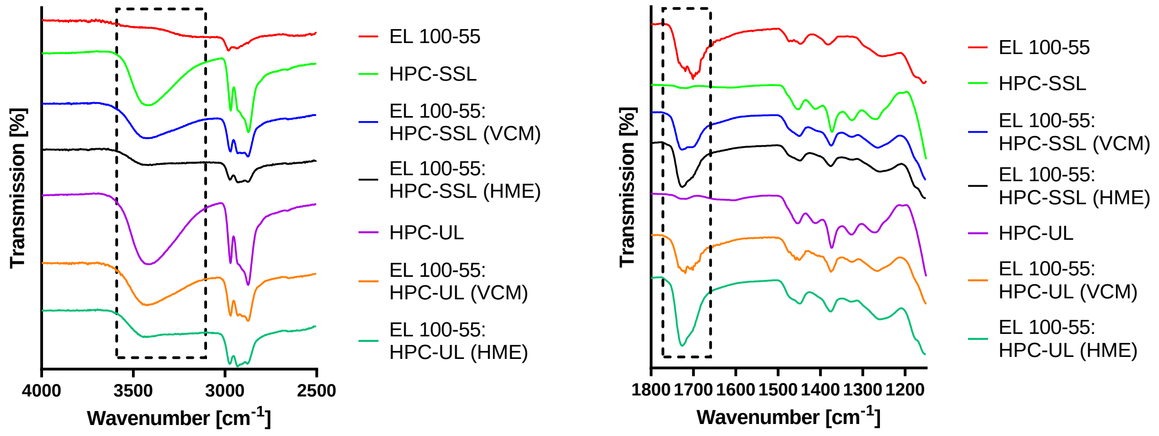

Fig. S3: Fourier-transform infrared (FT-IR) spectra of the single polymers and the polymer mixtures processed via vacuum compression molding (VCM) and hot-melt extrusion (HME).

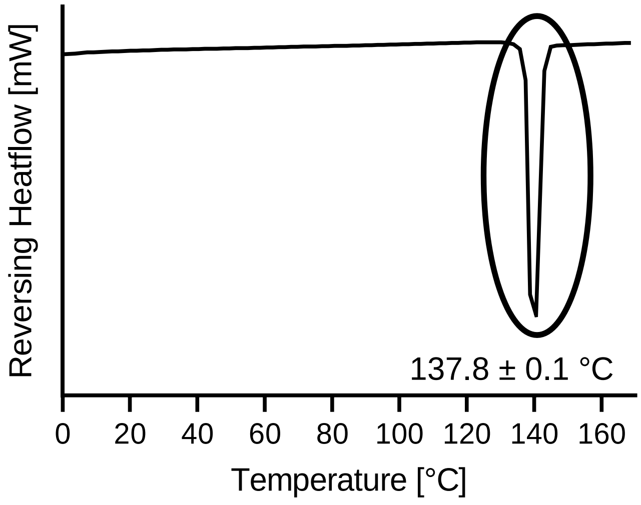

Fig. S4: Differential scanning calorimetry (DSC) thermograms (exo up) of neat EFV.

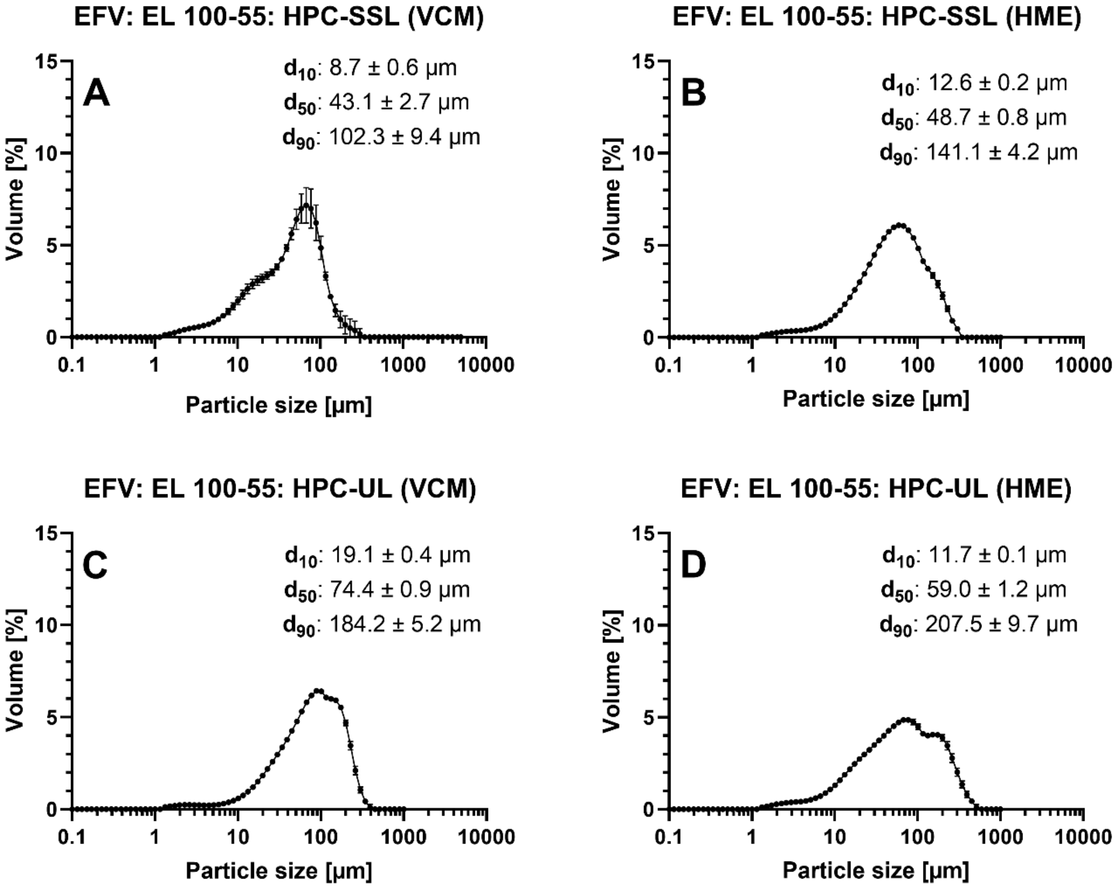

Fig. S5: Particle size distributions of investigated ternary ASDs (A, B, C, D), determined by a Horiba LA-960 laser diffractometer (Horiba, Kyoto, Japan) via a wet dispersion method. Milled particles were suspended under stirring into n-hexane with 0.1% Span® 80 and filled into a quartz cuvette. By utilizing a red laser diode with 650 nm wavelength (5 mW) and a blue light emitting diode with 405 nm wavelength (3 mW), particle sizes were measured.
